# Removal of Copper(II) from Aqueous Environment Using Silk Sericin-Derived Carbon

**DOI:** 10.3390/ijms231911202

**Published:** 2022-09-23

**Authors:** Yuting Xiao, Ruixiao Luo, Yansong Ji, Shiwei Li, Hongmei Hu, Xiaoning Zhang

**Affiliations:** 1 State Key Laboratory of Silkworm Genome Biology, College of Sericulture, Textile and Biomass Sciences, Southwest University, Chongqing 400715, China; 2 Westa College, Southwest University, Chongqing 400715, China; 3 Key Laboratory of Sustainable Utilization of Technology Research for Fisheries Resources of Zhejiang Province, Zhejiang Marine Fisheries Research Institute, Zhoushan 316021, China

**Keywords:** silk sericin, sericin-derived carbon, copper(II), adsorption

## Abstract

Sericin is a by-product of the silk industry. Its recycling contributes to environmental protection and the sustainable development of the cocoon silk industry. In this paper, on the basis of realizing sericin enrichment in solution, the Cu(II) adsorption capacities of sericin-derived carbon (SC), prepared at different pyrolysis temperatures, were studied. SC was characterized using scanning electron microscopy (SEM) and the zeta potential. The effects of the initial concentration of Cu(II), pH, adsorption temperature, and contact time on the adsorption process were evaluated, followed by an investigation of the mechanism of Cu(II) adsorption by SC. The results showed that SC has a porous structure that provides sites for Cu(II) adsorption. The maximum adsorption capacity of Cu(II) onto SC1050, 17.97 mg/g, was obtained at an adsorption temperature of 35 °C and a pH of 5.5. In addition, the pseudo-second-order kinetic model and Langmuir isotherm model correctly described the adsorption process of Cu(II) onto SC1050. Therefore, SC can act as a potential adsorbent for removing Cu(II) from water. This study helps promote the effective use of cocoon silk resources.

## 1. Introduction

Industries such as the electronics, brass manufacturing, fertilizer, pesticide, and textile industries are major sources of copper pollution in water [1,2,3]. Due to the non-biodegradability, persistence, extreme toxicity, and accumulation effect of Cu(II) [4], drainage of wastewaters without effective treatment can cause not only serious contamination of water resources, but also severe damage to the health of aquatic life, and from there to human beings. Relevant studies show that Cu(II) accumulation limits the growth and development of plants and causes a reduction in the fecundity of aquatic life [5,6]. Moreover, an enrichment of trace copper(II) in organisms can be achieved through the food web, leading to excessive intake of copper by people, which can evoke diarrhea, hepatic and renal dysfunctions, and severe mental and neurological illnesses [5,7,8,9]. Numerous approaches and techniques, including adsorption [10], membrane separation [11], electrocoagulation [12], chemical precipitation [13], and microbial treatment [14], have been used to remove copper ions from water. Compared with other technologies, adsorption has the advantages of simple operation, high efficiency, and relatively low cost, with a wide range of adsorbents [2,15,16,17,18,19].

Silk sericin (SS) is a highly hydrophilic macromolecular protein ranging from 20 and 400 kDa in molecular weight and composed of 18 amino acids [20]. It is a glue-like protein that coats silk fibroin fibers in raw silk offering protective and adhesive effects to the silk fibroin, and constituting 25–30% of the cocoon silk fibers [21]. To improve the luster, softness, and dyeability of silk fabrics, SS is traditionally discarded by a degumming process, resulting in much protein loss, which can further cause water contamination and eutrophication [22].

Several studies have worked on using discarded SS to remove heavy metal ions from water. Dusi et al. [23] reported the use of sericin–alginate particles to remove Cu(II) ions from aqueous solutions, and their maximum adsorption capacity was 87.27 mg/g. Singh et al. [24] showed that prepared chitosan–sericin conjugates could effectively remove Cr(VI) ions from water, with a maximum adsorption capacity of 139 mg/g. However, the above works did not discuss enriching SS from the aqueous environment. Furthermore, because of its hydrophilicity, SS from prepared composites can re-enter the water during the adsorption process.

Our previous work [25] reported that SS can be cross-linked with carboxymethyl chitosan (CMCS) through hydrogen bonding and then electrodeposited with CMCS on an anode to form composite hydrogel under low voltage. We proved that SS was the main component of SS/CMCS hydrogel, accounting for 77.12% of the hydrogel total mass.

Here, a prepared SS/CMCS hydrogel was pyrolyzed at 450 °C, 650 °C, 850 °C, and 1050 °C. Then, the fabricated hydrogel was applied to Cu(II) adsorption in water. The pyrolysis process effectively avoided the re-entry of SS into water during the adsorption.

## 2. Results and Discussion

### 2.1. Porous Structure Characterization

**Figure 1:**
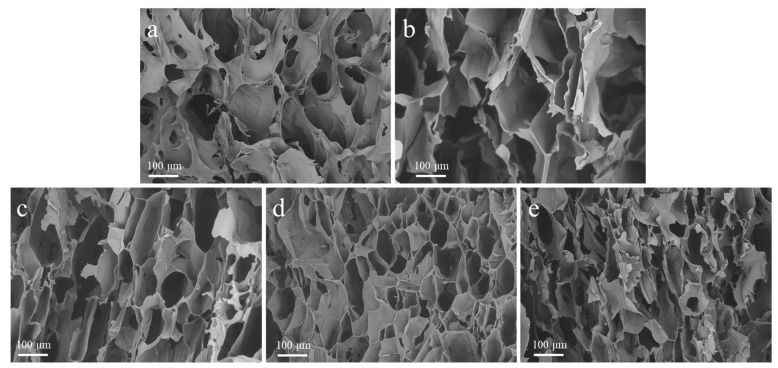
Representative SEM images of (**a**) SS/CMCS hydrogel, (**b**) SC450, (**c**) SC650, (**d**) SC850, and (**e**) SC1050.

Figure 1 shows the SEM images of freeze-dried hydrogel and the derived carbon pyrolyzed at different temperatures. As shown in Figure 1a, the SS/CMCS hydrogel surface exhibited an irregular, reticular, and porous structure, possibly due to intermolecular crosslinking among the CMCS and SS molecules [25]. Figure 1b–e shows that a higher pyrolysis temperature could lead to a more developed pore structure. As the pyrolysis temperature increased from 450 °C to 1050 °C, the pore size on the surface of SC450, SC650, SC850, and SC1050 gradually shrank and the pore structure became densely arranged, which was consistent with a previous report that the carbon framework continued to shrink as the temperature of heat treatment gradually increased in [26]. Table 1 shows that the BET surface area of each sample gradually increased with the carbonization temperature, providing a larger contact area and more adsorption sites, thus improving the adsorption capacity of SC [27].

### 2.2. Batch Adsorption Experiments

#### 2.2.1. Effect of Initial Concentration

**Figure 2:**
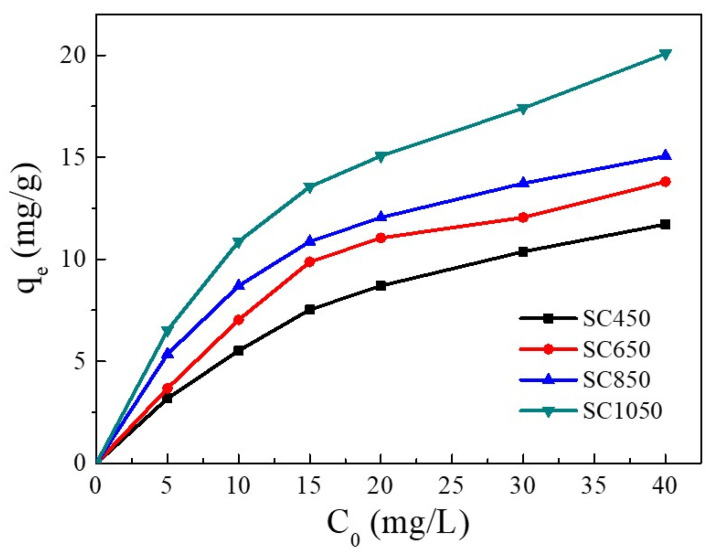
Influence of Cu(II) initial concentration on the adsorption capacity (pH = 5.0, adsorption temperature = 25 °C, contact time = 24 h).

The influence of Cu(II) initial concentration on the adsorption capacity is shown in Figure 2. Increasing Cu(II) initial concentration enhanced the adsorption capacity of Cu(II) on all SC samples. With the Cu(II) initial concentration increasing from 5 mg/L to 40 mg/L, the adsorption capacity of Cu(II) on SC450, SC650, SC850, and SC1050 increased gradually. This result was due to the increase in Cu(II) initial concentration enhancing the driving force of adsorption processes, thus enhancing the removal of Cu(II) ions from the solution [28,29].

On the other hand, SC had a higher absorption capacity when pyrolyzed at 1050 °C instead of 450 °C, 650 °C, and 850 °C. At a Cu(II) solution concentration of 40 mg/L, the equilibrium adsorption amount of SC450, SC650, and SC850 for Cu(II) was 11.71 mg/g, 13.80 mg/g, and 15.06 mg/g, respectively. Increasing the pyrolysis temperature to 1050 °C increased the adsorption amount of Cu(II) on SC1050 to 20.08 mg/g. SEM and BET results showed that the SC prepared at 1050 °C had a denser pore structure, which led to a larger SC1050 surface area, provided more adsorption sites, and thus increased the adsorption capacity of Cu(II) on SC1050 [30]. Therefore, SC1050 was used as the adsorbent in subsequent experiments.

#### 2.2.2. Effect of pH

**Figure 3:**
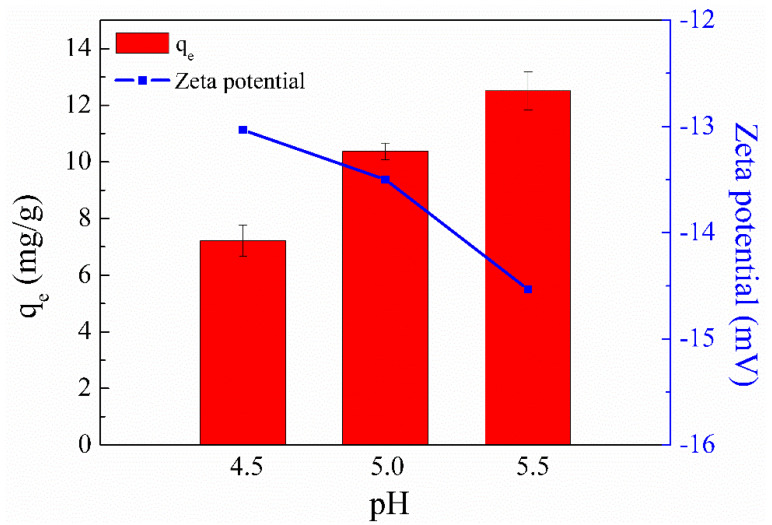
Effect of solution pH on the Cu(II) adsorption capacity of SC1050 and the zeta potential of SC1050 at three pH values (Cu(II) initial concentration = 15 mg/L, adsorption temperature = 25 °C, contact time = 24 h).

The initial solution pH is crucial for the metal ion adsorption capacity of an adsorbent because it can affect not only the surface charge and ionization degree of the adsorbent but also the aqueous solution chemistry of metal ions [31]. Since turbidity (suspensions of copper hydroxide) could be clearly observed in Cu(II) solution when the solution pH was adjusted to 6 [32], a pH range of 4.5–5.5 was selected to explore the influence of pH on Cu(II) adsorption. Figure 3 shows that SC1050 exhibited the lowest Cu(II) ion adsorption capacities when the solution pH was 4.5. This result could be attributed to a large amount of H^+^ ions in the solution competing with Cu^2+^ ions for the active adsorption sites, resulting in low adsorption capacity of Cu(II) ions [33].

In addition, the zeta potential of SC1050 at pH values of 4.5, 5.0, and 5.5 was measured to explore its adsorption mechanism. Figure 3 shows that a solution pH of 5.5 made the surface charge of SC1050 more negative, increasing the ionic strength between SC1050 and the positively charged Cu(II) ions, which made the adsorption more effective. Therefore, a pH of 5.5 was selected for subsequent adsorption experiments.

#### 2.2.3. Effect of Temperature on the Sorption of Cu(II) Ions and a Thermodynamic Study

**Figure 4:**
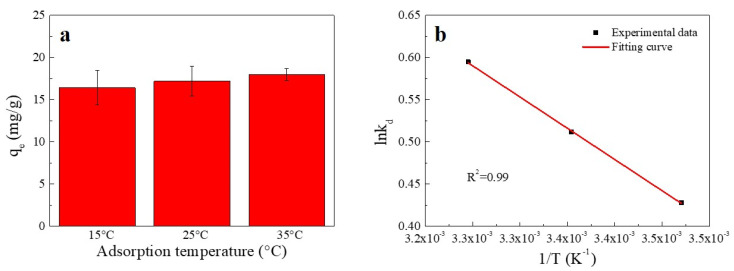
(**a**) Effect of temperature on Cu(II) adsorption onto SC1050 and (**b**) a van’t Hoff plot for adsorption of Cu(II) onto SC1050 (Cu(II) initial concentration = 15 mg/L, pH = 5.5, contact time = 24 h).

The influence of temperature on the sorption of Cu(II) is shown in Figure 4. An increase in temperature was found to enhance the sorption of Cu(II) onto SC1050. This enhancement might have been due to the higher temperature providing sufficient energy for copper ions to bind to the surface and interior of SC1050 [2].

The thermodynamic fitting curve is shown in Figure 4b, and the thermodynamic parameters are reported in Table 2. According to Table 2, the negative ΔG value increased with increasing temperature, showing that the adsorption process was more favorable at higher temperatures and implying that the adsorption process was chemisorption [9]. In addition, the positive ΔH value indicated that adsorption was endothermic. Moreover, as the value of ΔS was positive, the probability of a successful collision between Cu(II) and SC1050 might increase, which could strengthen the Cu(II) adsorption [33].

#### 2.2.4. Effect of Contact Time on the Adsorption Process and the Adsorption Kinetics

**Figure 5:**
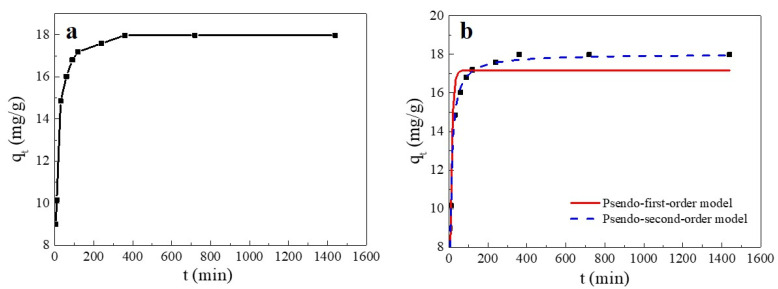
(**a**) Effect of contact time on the Cu(II) adsorption capacity of SC1050 and (**b**) pseudo-first-order and pseudo-second-order plots of Cu(II) sorption onto SC1050 (Cu(II) initial concentration = 15 mg/L, pH = 5.5, adsorption temperature = 35 °C).

As shown in Figure 5, the adsorption amount of Cu(II) on SC1050 varied with contact time and could be divided into three stages: the adsorption rate was fast within the initial 30 min, it gradually slowed down from 30 min to 6 h, and the adsorption reached equilibrium at 6 h. The explanation was that in the early stage of the adsorption process, many adsorption sites on the SC1050 surface were rapidly occupied by Cu(II) ions, resulting in a fast adsorption rate [34]. However, the adsorption sites progressively became saturated over time and reached equilibrium.

To explore the adsorption process kinetics, pseudo-first-order and pseudo-second-order kinetic models were used to fit the experimental results. The fitting curve and the calculated parameters are reported in Figure 5 and Table 3, respectively. The adsorption appeared to fit better to a pseudo-second-order kinetic model, indicating that the adsorption process of SC1050 was mainly controlled by chemisorption, which involved the sharing or exchange of electrons between Cu(II) and SC1050 [35].

**Figure 6:**
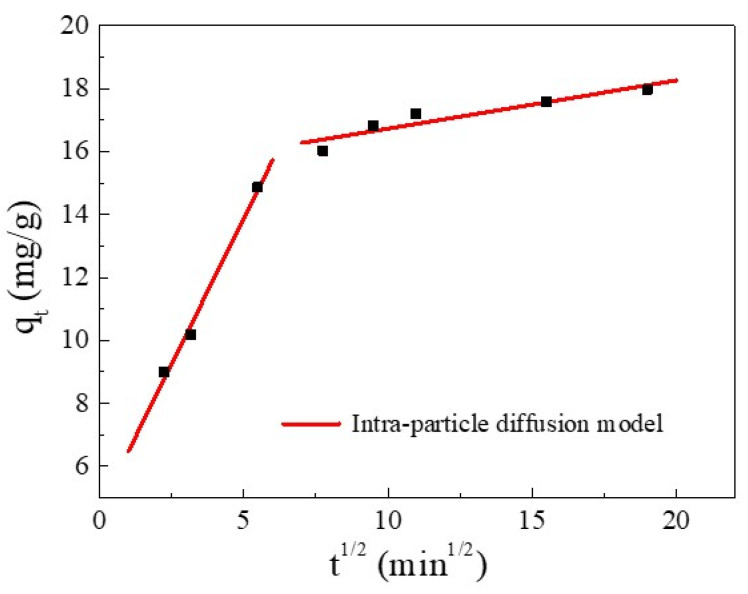
The IPD model for Cu(II) adsorption.

Furthermore, the kinetics and rate-limiting step of the adsorption were elucidated using the intra-particle diffusion (IPD) model. The IPD fitting curve of Cu(II) adsorption and the values of the rate constant are reported in Figure 6 and Table 4, respectively. Figure 6 shows that the plot exhibited a multi-linearity, in accordance with two stages: the first linear portion was due to the diffusion of Cu(II) through the solution to the external surface of SC1050 and the second linear portion described intra-particle diffusion [36]. The rate constant of the first stage was greater than that of the second stage (Table 4), indicating the initial adsorption was rapid surface adsorption controlled by film diffusion, and intra-particle diffusion controlled the adsorption process [35]. Additionally, the fitting plot did not intersect the origin, indicating that intra-particle diffusion was not the only rate-limiting step [33].

#### 2.2.5. Adsorption Isotherm

**Figure 7:**
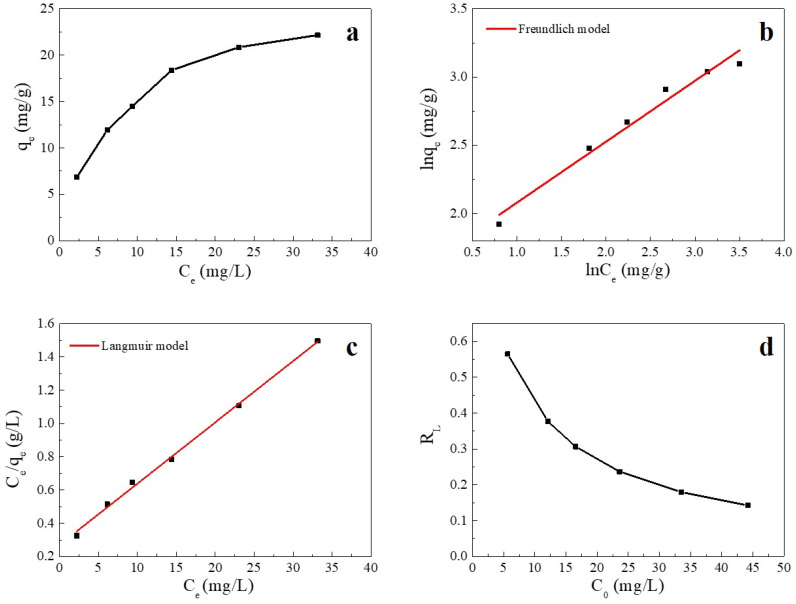
(**a**) Adsorption equilibrium isotherm of Cu(II) on SC1050, (**b**) the fitting plot of the Langmuir isotherm, (**c**) the fitting plot of the Freundlich isotherm, and (**d**) a plot of the separation factor versus initial Cu(II) concentration (pH = 5.5, adsorption temperature = 35 °C, contact time = 24 h).

Adsorption isotherms are used to describe the relationship between the adsorbate and adsorbent at equilibrium [37]. The fitting plots and fitting parameters of Cu(II) adsorption onto SC1050 are shown in Figure 7 and Table 5, respectively, which reveal that the Langmuir model could better describe the process of Cu(II) adsorption onto SC1050 at 35 °C, indicating monolayer adsorption of Cu(II) ions onto SC1050. Furthermore, Figure 7d shows that the R_L_ values calculated by the Langmuir model were greater than 0 but less than 1 for all initial concentrations, meaning that the adsorption process of Cu(II) onto SC1050 was favorable [1].

### 2.3. Determination of Cu on SC Using ICP-OES

SC1050 was placed in 15 mg/L of Cu(II) solution for 24 h (experimental group). Then, ICP-OES was used to determine the Cu on SC1050. According to Table 6, an enrichment of Cu on SC1050 was observed, compared with the control group (no immersion in Cu solution).

## 3. Materials and Methods

### 3.1. Chemicals

CMCS was purchased from Shanghai Ryon Biological Technology Co., Ltd. (Shanghai, China). Sodium chloride was purchased from Aladdin Biotech Co., Ltd. (Shanghai, China). Copper nitrate hydrate and hydroxylammonium chloride were purchased from Shanghai Macklin Biochemical Co., Ltd. (Shanghai, China). Trisodium citrate dihydrate and sodium acetate trihydrate were purchased from Chengdu Chron Chemicals Co., Ltd. (Chengdu, China). Acetic acid was purchased from Sangon Biotech Co., Ltd. (Shanghai, China). The 2,9-Dimethyl-1,10-phenanthroline hemihydrate was purchased from Tianjin Heowns Biochemical Technology Co., Ltd. (Tianjin, China). Nitric acid, hydrofluoric acid, hydrochloric acid, and hydrogen peroxide were purchased from the Beijing Institute of Chemical Reagents (Beijing, China). Deionized water was from a Milli-Q Direct-8 purification system (resistivity >18 MΩ·cm, Millipore Corp., Boston, MA, USA).

### 3.2. Sample Preparation

#### 3.2.1. Fabrication of the SS/CMCS Hydrogel

A CMCS solution with a concentration of 1% (*w*/*v*) was prepared and adjusted to pH = 12 with 5 M NaOH to ensure that the CMCS could be completely dissolved [38]. Then, SS powder was dissolved in deionized water to prepare an SS solution with a concentration of 8% (*w*/*v*). The SS and CMCS solutions were mixed in equal volumes at room temperature, and the resulting solution was adjusted to pH = 7.5 with 0.1 M NaOH. After magnetic stirring for 4 h, the SS/CMCS solution was stored at 4 °C overnight for later use.

A three-electrode assembly was immersed in 20 mL of the SS/CMCS mixture containing 5 mg of NaCl as the electrolyte, and the SS/CMCS hydrogel was fabricated using an electrochemical workstation (CHI760E, Shanghai Chenhua Instruments Co., Ltd., Shanghai, China), according to the method described in our previous work [25]. The hydrogel generated on the working electrode was transferred to deionized water and rinsed for 15 min to remove any residue. This rinsing process was repeated three times.

#### 3.2.2. Preparation of Sericin-Based Carbon

The prepared SS/CMCS hydrogel was placed in a −20 °C refrigerator for 4 h and then transferred to a −80 °C refrigerator for preservation. After 8 h, the frozen SS/CMCS hydrogel was freeze-dried using a lyophilizer (LGJ-10, YuMing Instrument Co., Ltd., Shanghai, China), and then the freeze-dried hydrogel was pyrolyzed. Adopting a carbonization procedure, similar to that of our previously reported work [25], we placed the sample in a tube furnace (OTF-1200X-S, Hefei KeJing Materials Technology Co., Ltd., Hefei, China) and maintained it for 120 min after the temperature reached 450 °C, 650 °C, 850 °C, and 1050 °C, then cooled it to ambient temperature. Subsequently, the sample was rinsed with deionized water at 60 °C every 30 min until the pH of the rinse water was neutral, and, then, the sample was freeze-dried to remove water. Finally, the SC was obtained. For simplicity, SCs obtained at different pyrolysis temperatures were labeled as SC450, SC650, SC850, and SC1050, respectively, and stored in desiccators for later use.

### 3.3. SC Characterization

The relationships between the mass and temperature change in the freeze-dried SS solution, CMCS hydrogel, and SS/CMCS hydrogel were investigated using a thermo-gravimetric analyzer (STA 449 F3 Jupiter, Netzsch, Germany). The samples were heated from 30 °C to 800 °C at a heating rate of 10 °C/min under a constant N_2_ flow. The micromorphology of the SS/CMCS hydrogel and its derived carbon prepared at different temperatures was observed using scanning electron microscopy (SEM, TM4000Plus, Tokyo, Japan). The specific surface area was estimated by the Brunauer–Emmett–Teller (BET) method through a N_2_ adsorption–desorption test at 77 K (ASAP 2460, Micromeritics, Norcross, GA, USA). The zeta potential of SC1050 under different pH values was measured by a Zetasizer Nano ZS90 (Malvern Instruments, Worcestershire, UK).

### 3.4. Batch Adsorption Experiment

The effects of Cu(II) initial concentration, solution pH, adsorption temperature, and contact time on Cu(II) removal were investigated. A certain mass of copper nitrate was dissolved in deionized water to obtain a Cu(II) stock solution of 1000 mg/L, which was then diluted to the corresponding concentration in subsequent experimental use.

#### 3.4.1. Effect of Cu(II) Initial Concentration

The 1000 mg/L Cu(II) stock solution was diluted into 6 groups with initial concentrations of 5, 10, 15, 20, 30, and 40 mg/L, and the initial solution pH was adjusted to 5.0. The ratio of the SC sample mass to the Cu(II) solution volume in the weighing bottle was 0.5 g/L. The weighing bottles were placed in a shaking incubator (LYGZ-2102C, JTLIANGYOU Instrument Co., Ltd., Jiangsu, China) at a speed of 100 rpm at 25 °C for 24 h. The filtrate was obtained after filtration through a 0.22 μm filter membrane (Jiangsu Green Union Science Instrument Co., Ltd., Jiangsu, China). The concentration of Cu(II) in the filtrate after adsorption was determined according to Chinese Standard HJ 486-2009 (water quality determination of copper-2,9-dimethyl-1,10-phenanthroline spectrophotometric method) [39]. Briefly, the Cu(II) ion was reduced by hydroxylamine hydrochloride to its cuprous form, which, in turn, was reacted with 2,9-dimethyl-1,10-phenanthroline to form a yellow complex. This complex was then measured via an ultraviolet-visible (UV–Vis) spectrophotometer (UV1900, Shanghai Jinghua Technology Instrument Co., Ltd., Shanghai, China) at 457 nm. To ensure experimental accuracy, all experiments were performed in triplicate.

The equilibrium adsorption amount *q_e_* (mg/g) and the percentage removal *R* (%) were calculated by Equations (1) and (2), respectively [40]:

(1) $$q_{e}=\frac{(C_{0}-C_{e})\;\times \;V}{M}$$

(2) $$R\;\%=\frac{C_{0}-C_{e}}{C_{0}}\times 100\%$$

where *C*_0_ and *C_e_* are the concentrations (mg/L) of Cu(II) in solution at the initial and equilibrium states, *M* is the SC weight (g), and *V* is the volume of Cu(II) solution (mL).

#### 3.4.2. Effect of Initial pH

Cu(II) solution with a concentration of 15 mg/L was selected, and the initial solution pH was adjusted to 4.5, 5.0, and 5.5. The mass-to-volume ratio of the SC sample and Cu(II) solution in the weighing bottles was maintained at 0.5 g/L. The weighing bottles were placed in a shaking incubator at a speed of 100 rpm at 25 °C for 24 h. After filtration, the Cu(II) solution concentrations were determined according to Chinese Standard HJ 486-2009 [39].

#### 3.4.3. Effect of Adsorption Temperature and a Thermodynamic Study

Cu(II) solution with a concentration of 15 mg/L was selected, and the initial solution pH was adjusted to 5.5. The mass-to-volume ratio of the SC sample and Cu(II) solutions in the weighing bottle was maintained at 0.5 g/L. The weighing bottles were placed in a shaking incubator with a rotational speed of 100 rpm for 24 h at different temperatures (15 °C, 25 °C, and 35 °C). After filtration, the Cu(II) solution concentrations were determined according to Chinese Standard HJ 486-2009 [39].

The Gibbs free energy change (Δ*G*, kJ/mol), the enthalpy change (Δ*H*, kJ/mol), and the entropy change (Δ*S*, J/mol·K) were calculated by the van’t Hoff equation to investigate the thermodynamic behavior of Cu(Ⅱ) adsorption onto SC1050. The equations are shown in (3)–(5) [9]:

(3) $$k_{d}=\frac{q_{e}}{C_{e}}$$

(4) $$\ln k_{d}=\frac{-\Delta H}{\mathrm{RT}}+\frac{\Delta S}{R}$$

(5) $$\Delta G=-RT\;ln\;k_{d}$$

where *T* is the absolute temperature (K), and *R* is the gas constant (8.314 J/mol·K).

#### 3.4.4. Effect of Contact Time and Adsorption Kinetics

Cu(II) solution with a concentration of 15 mg/L was selected, and the initial solution pH was adjusted to 5.5. The mass-to-volume ratio of the SC sample and Cu(II) solution in the weighing bottle was maintained at 0.5 g/L. The weighing bottles were placed in a shaking incubator at 35 °C with a rotational speed of 100 rpm to oscillate for 5 min, 10 min, 30 min, 60 min, 90 min, 120 min, 240 min, 360 min, 720 min, and 1440 min. After filtration, the Cu(II) solution concentrations were determined according to Chinese Standard HJ 486-2009 [39].

The amount of Cu(II) adsorbed at time *t*, *q_t_* (mg/g), can be calculated through Equation (6):

(6) $$q_{t}=\frac{(C_{0}-C_{t})\;\times \;V}{M}$$

To explore the kinetics of the adsorption process of Cu(II) onto SC1050, pseudo-first-order kinetics, pseudo-second-order kinetics, and the intra-particle diffusion model were selected to fit the experimental data

The pseudo-first-order kinetic model is shown in Equation (7) [27]:

(7) $$q_{t}=q_{e}\left({1\;-\;e}^{-k_{1}t}\right)$$

where *k*_1_ is the pseudo-first-order adsorption rate constant (L/min), and *t* is the adsorption time (min).

The pseudo-second-order kinetic model is shown in Equation (8) [35]:

(8) $$q_{t}=\frac{k_{2}q_{e}^{2}t}{1+k_{2}q_{e}t}$$

where *k*_2_ is the pseudo-second-order rate constant (g·mg^−1^·min^−1^) and *t* is the adsorption time (min).

The intra-particle diffusion (IPD) model is shown in Equation (9) [35]:

(9) $$q_{t}=k_{i}t^{1/2}+C$$

where *k_i_* is the diffusion coefficient of the particle (mg·g^−1^·min^−1/2^), and *C* is a constant. A larger *C* indicates a stronger effect of the boundary layer on the adsorption process [33].

#### 3.4.5. Adsorption Isotherm

To investigate the maximum adsorption capacity of the adsorbent, Cu(II) stock solution of 1000 mg/L was diluted to 5, 10, 15, 20, 30, and 40 mg/L. The initial solution pH was then adjusted to 5.5. The mass-to-volume ratio of the SC sample and Cu(II) solution in the weighing bottle was maintained at 0.5 g/L. The weighing bottles were then placed in a shaking incubator operating at a rotational speed of 100 rpm at 35 °C for 24 h. After filtration, the Cu(II) solution concentrations were determined according to Chinese Standard HJ 486-2009 [39].

Langmuir and Freundlich isotherm models were applied to modeling the adsorption isotherms. The Langmuir isotherm model for monolayer adsorption on homogeneous sites is described by Equation (10) [2]:

(10) $$\frac{C_{e}}{q_{e}}=\frac{1}{q_{m}\cdot k_{L}}+\frac{C_{e}}{q_{m}}$$

where *q_m_* is the maximum adsorption amount at equilibrium (mg/g), and *k_L_* is the Langmuir isotherm constant associated with the adsorption free energy (L/mg).

The dimensionless separation factor *R_L_* is the basic characteristic of a Langmuir isotherm and can be used to determine the favorability of an adsorption process. It is calculated by Equation (11) [1]:

(11) $$R_{L}=\frac{1}{1+k_{L}C_{0}}$$

where *k_L_* is the Langmuir isotherm constant. The adsorption is favorable when 0 < *R_L_* < 1, unfavorable when *R_L_* > 1, and irreversible when *R_L_* = 0.

The Freundlich isotherm model for multilayer adsorption on heterogeneous sites is expressed as Equation (12) [2]:

(12) $$ln\;q_{e}=ln\;k_{F}+\frac{1}{n}\cdot ln\;C_{e}$$

where *n* is the Freundlich constant related to surface heterogeneity.

### 3.5. Adsorption of Cu(II) Determined by Inductively Coupled Plasma Optical Emission Spectrometry

The sample was carefully weighed and then mixed with 5 mL of HNO_3_, 1 mL of HF, 1 mL of H_2_O_2_, and 1 mL of HCl. The mixture was placed in an autoclave at 180 °C for 8 h. Then, the reactor was cooled to room temperature. Finally, the mixture inside was transferred into 25 mL volumetric flasks and made up to the volume for inductively coupled plasma optical emission spectrometry (ICP-OES; Agilent 730, Agilent Technologies, Santa Clara, CA, USA) analysis.

### 3.6. Data Analysis

All adsorption experiments were repeated 3 times. Microsoft Excel 2019 (Redmond, WA, USA) and Origin software (Origin Pro 8.0, OriginLab Corp., Northampton, MA, USA) were used for data analysis and graphing.

## 4. Conclusions

In this study, the adsorption capacity of carbon derived from SS for Cu(II) was investigated. An SS/CMCS hydrogel with porosity was fabricated by electrodeposition. The SC samples were further fabricated by pyrolysis at 450 °C, 650 °C, 850 °C, and 1050 °C. SEM results indicated that SC1050 had a more developed pore structure, which increased the surface area and provided more adsorption sites for Cu(II) adsorption.

The results of batch adsorption experiments showed that SC1050 had the highest adsorption capacity and removal rate of Cu(II), and the optimum condition for Cu(II) adsorption by SC1050 was T = 35 °C and pH = 5.5. The adsorption of Cu(II) by SC1050 was a spontaneous, endothermic process. The fitted Cu(II) adsorption results agreed with the pseudo-second-order kinetic model and the Langmuir isotherm. The maximum adsorption amount of Cu(II) on SC1050 was 17.97 mg/g. Compared with pistachio green hull-derived carbon (19.84 mg/g) [41], apple tree branch-derived carbon (11.41 mg/g) [33], and *Chaenomeles Sinensis* seed-derived carbon (15.78 mg/g) [27], SC1050 showed a better Cu(II) adsorption capacity. Therefore, SC1050 can act as an effective adsorbent for removing Cu(II) from aqueous solution.

## Figures and Tables

**Table 1:** BET surface area of the samples.

Sample	BET Surface Area (m^2^/g)
SC450	146.27
SC650	632.30
SC850	1036.87
SC1050	1340.45

**Table 2:** Thermodynamic parameters for adsorption of Cu(II) onto SC1050.

T (K)	ΔG (kJ/mol)	ΔH (kJ/mol)	ΔS (J/mol·K)
288.15	−1.02	6.16	24.91
298.15	−1.27		
308.15	−1.52		

**Table 3:** Pseudo-first-order, pseudo-second-order, and IPD models of Cu(II) adsorption onto SC1050.

Sample	q_e,exp_ (mg/g)	Pseudo-First-Order			Pseudo-Second-Order		
		q_e,cal_ (mg/g)	k_1_ × 10^−^^3^ (min^−^^1^)	R^2^	q_e,cal_ (mg/g)	k_2_ × 10^−^^3^ (g/mg·min)	R^2^
SC1050	17.98	17.17	0.11	0.87	18.02	0.01	0.97

**Table 4:** Intra-particle diffusion rate constant for the adsorption of Cu(II) onto SC1050.

Sample	IPD	
	k_i1_ (mg/g·min^1/2^)	k_i2_ (mg/g·min^1/2^)
SC1050	1.85	0.15

**Table 5:** Langmuir and Freundlich isotherm parameters for Cu(II) adsorption onto SC1050.

Sample	Langmuir Isotherm Model			Freundlich Isotherm Model		
	q_max,cal_ (mg/g)	k_L_ (L/mg)	R^2^	n	k_F_ (L/mg)	R^2^
SC1050	27.14	0.11	0.87	18.02	0.01	0.97

**Table 6:** Cu content in SC1050.

Group	Cu Content (mg/L)
Control	0.012 ± 0.017
Experimental	0.212 ± 0.007

## Data Availability

Data are contained within the article.

## References

[1] Tomczyk A., Sokołowska Z., Boguta P. (2020). Biomass type effect on biochar surface characteristic and adsorption capacity relative to silver and copper. Fuel.

[2] Cao Q.Y., Huang Z.H., Liu S.G., Wu Y.P. (2019). Potential of punica granatum biochar to adsorb Cu(II) in soil. Sci. Rep..

[3] Zhang X.X., Shi X.J., Ma L., Pang X., Li L.L. (2019). Preparation of chitosan stacking membranes for adsorption of copper ions. Polymers.

[4] Zhang P.Z., Zhang X.X., Yuan X.R., Xie R.Y., Han L.J. (2021). Characteristics, adsorption behaviors, Cu(II) adsorption mechanisms by cow manure biochar derived at various pyrolysis temperatures. Bioresour. Technol..

[5] Tomczyk A., Sokolowska Z., Boguta P., Szewczuk-Karpisz K. (2020). Comparison of monovalent and divalent ions removal from aqueous solutions using agricultural waste biochars prepared at different temperatures-experimental and model study. Int. J. Mol. Sci..

[6] Cairns S., Robertson I., Sigmund G., Street-Perrott A. (2020). The removal of lead, copper, zinc and cadmium from aqueous solution by biochar and amended biochars. Environ. Sci. Pollut. Res..

[7] Abu El-Soad A.M., Lazzara G., Abd El-Magied M.O., Cavallaro G., Al-Otaibi J.S., Sayyed M.I., Kovaleva E.G. (2022). Chitosan functionalized with carboxyl groups as a recyclable biomaterial for the adsorption of Cu (II) and Zn (II) ions in aqueous media. Int. J. Mol. Sci..

[8] Xie X.Y., Deng R.K., Pang Y.Q., Bai Y., Zheng W.J., Zhou Y.H. (2017). Adsorption of copper(II) by sulfur microparticles. Chem. Eng. J..

[9] Wu Q.L., Dong S.Z., Wang L.J., Li X.Y. (2021). Single and competitive adsorption behaviors of Cu^2+^, Pb^2+^ and Zn^2+^ on the biochar and magnetic biochar of pomelo peel in aqueous solution. Water.

[10] Liu L.Q., Huang Y.J., Meng Y.H., Gao J.H., Hu H.J., Su Y.H., Dong L., Tao S.N., Ruan R. (2020). Investigating the adsorption behavior and quantitative contribution of Pb^2+^ adsorption mechanisms on biochars by different feedstocks from a fluidized bed pyrolysis system. Environ. Res..

[11] Hao S., Jia Z.Q., Wen J.P., Li S.D., Peng W.J., Huang R.Y., Xu X. (2020). Progress in adsorptive membranes for separation—A review. Sep. Purif. Technol..

[12] Ithan F., Ulucan-Altuntas K., Avsar Y., Kurt U., Saral A. (2019). Electrocoagulation process for the treatment of metal-plating wastewater: Kinetic modeling and energy consumption. Front. Environ. Sci. Eng..

[13] Zeng Z., Zheng P., Kang D., Li Y.Y., Li W.J., Xu D.D., Chen W.D., Pan C. (2020). The removal of copper and zinc from swine wastewater by anaerobic biological-chemical process: Performance and mechanism. J. Hazard. Mater..

[14] Zhang Q.Q., Ji X.M., Tian G.M., Jin R.C. (2020). Evolution of microbial community and antibiotic resistance genes in anammox process stressed by oxytetracycline and copper. Bioresour. Technol..

[15] Akhmetzhan A., Myrzakhmetova N., Amangeldi N., Kuanyshova Z., Akimbayeva N., Dosmaganbetova S., Toktarbay Z., Longinos S.N. (2021). A short review on the N,N-Dimethylacrylamide-based hydrogels. Gels.

[16] Azat S., Arkhangclsky E., Papathanasiou T., Zorpos A., Abirov A., Inglezkis V.J. (2020). Synthesis of biosourced silica–Ag nanocomposites and amalgamation reaction with mercury in aqueous solutions. Comptes Rendus. Chim..

[17] Akhmetzhan A., Abeu N., Longinos S.N., Tashenov A., Myrzakhmetova N., Amangeldi N., Kuanyshova Z., Ospanova Z., Toktarbay Z. (2021). Synthesis and heavy-metal sorption studies of N,N-Dimethylacrylamide-based hydrogels. Polymers.

[18] Mahmoodi N.M., Taghizadeh M., Taghizadeh A. (2018). Mesoporous activated carbons of low-cost agricultural bio-wastes with high adsorption capacity: Preparation and artificial neural network modeling of dye removal from single and multicomponent (binary and ternary) systems. J. Mol. Liq..

[19] Zhang Z., Wang T., Zhang H., Liu Y., Xing B. (2021). Adsorption of Pb(II) and Cd(II) by magnetic activated carbon and its mechanism. Sci. Total Environ..

[20] Kunz R.I., Costa Brancalhao R.M., De Chasko Ribeiro L.F., Marcal Natali M.R. (2016). Silkworm sericin: Properties and biomedical applications. BioMed Res. Int..

[21] Yamano M., Hirose R., Lye P.Y., Takaki K., Maruta R., On Liew M.W., Sakurai S., Mori H., Kotani E. (2022). Bioengineered silkworm for producing cocoons with high fibroin content for regenerated fibroin biomaterial-based applications. Int. J. Mol. Sci..

[22] Lamboni L., Gauthier M., Yang G., Wang Q. (2015). Silk sericin: A versatile material for tissue engineering and drug delivery. Biotechnol. Adv..

[23] Dusi G.G., Marques G.S., Kienteca M.L., Gimenes M.L., Cerutti M.L.M.N., Da Silva V.R. (2022). Biosorption investigation of Cu(II) ions from aqueous solutions using sericin–alginate particles: Kinetic, equilibrium, and thermodynamic. Sustain. Chem. Pharm..

[24] Singh S.P., Rathinam K., Kasher R., Arnusch C.J. (2018). Hexavalent chromium ion and methyl orange dye uptake via a silk protein sericin–chitosan conjugate. RSC Adv..

[25] Ji Y.S., Zhang X.N., Chen Z.Y., Xiao Y.T., Li S.W., Gu J., Hu H.M., Cheng G.T. (2022). Silk sericin enrichment through electrodeposition and carbonous materials for the removal of methylene blue from aqueous solution. Int. J. Mol. Sci..

[26] Lv Y., Zhang F., Dou Y., Zhai Y., Wang J., Liu H., Xia Y., Tu B., Zhao D. (2012). A comprehensive study on KOH activation of ordered mesoporous carbons and their supercapacitor application. J. Mater. Chem..

[27] Hu X.L., Song J.Y., Wang H.Y., Zhang W., Wang B., Lyu W.L., Wang Q.L., Liu P., Chen L., Xing J. (2019). Adsorption of Cr(VI) and Cu(II) from aqueous solutions by biochar derived from Chaenomeles sinensis seed. Water Sci. Technol..

[28] Herath G.A.D., Poh L.S., Ng W.J. (2019). Statistical optimization of glyphosate adsorption by biochar and activated carbon with response surface methodology. Chemosphere.

[29] Allen S., Mckay G., Khader K. (1989). Intraparticle diffusion of a basic dye during adsorption onto sphagnum peat. Environ. Pollut..

[30] Stirling R.J., Snape C.E., Meredith W. (2018). The impact of hydrothermal carbonisation on the char reactivity of biomass. Fuel Process. Technol..

[31] Wang Q., Wang B., Lee X.Q., Lehmann J., Gao B. (2018). Sorption and desorption of Pb(II) to biochar as affected by oxidation and pH. Sci. Total Environ..

[32] Meng J., Feng X., Dai Z., Liu X., Wu J., Xu J. (2014). Adsorption characteristics of Cu(II) from aqueous solution onto biochar derived from swine manure. Environ. Sci. Pollut. Res. Int..

[33] Zhao S.X., Ta N., Wang X.D. (2020). Absorption of Cu(II) and Zn(II) from aqueous solutions onto biochars derived from apple tree branches. Energies.

[34] Lou K.Y., Rajapaksha A.U., Ok Y.S., Chang S.X. (2016). Sorption of copper(II) from synthetic oil sands process-affected water (OSPW) by pine sawdust biochars: Effects of pyrolysis temperature and steam activation. J. Soils Sediments.

[35] Choudhary M., Kumar R., Neogi S. (2020). Activated biochar derived from opuntia ficus-indica for the efficient adsorption of malachite green dye, Cu^2+^ and Ni^2+^ from water. J. Hazard. Mater..

[36] Zhao S., Zhan Y., Wan X., He S., Yang X., Hu J., Zhang G. (2020). Selective and efficient adsorption of anionic dyes by core/shell magnetic MWCNTs nano-hybrid constructed through facial polydopamine tailored graft polymerization: Insight of adsorption mechanism, kinetic, isotherm and thermodynamic study. J. Mol. Liq..

[37] Mall I.D., Srivastava V.C., Agarwal N.K., Mishra I.M. (2005). Removal of congo red from aqueous solution by bagasse fly ash and activated carbon: Kinetic study and equilibrium isotherm analyses. Chemosphere.

[38] Chen Z.Y., Zhang X.N., Liang J.W., Ji Y.S., Zhou Y.Q., Fang H. (2021). Preparation of silk fibroin/carboxymethyl chitosan hydrogel under low voltage as a wound dressing. Int. J. Mol. Sci..

[39] (2009). National Standard of the People’s Republic of China. Water Quality—Determination of Copper-2,9-Dimethyl-1,10-phenanthroline Spectrophotometric Method.

[40] Mo G.H., Xiao J., Gao X. (2022). To enhance the Cd^2+^ adsorption capacity on coconut shell-derived biochar by chitosan modifying: Performance and mechanism. Biomass Convers. Biorefin..

[41] Jalayeri H., Pepe F. (2019). Novel and high-performance biochar derived from pistachio green hull biomass: Production, characterization, and application to Cu(II) removal from aqueous solutions. Ecotox. Environ. Safe..

